# A Web-Based and Print-Delivered Computer-Tailored Physical Activity Intervention for Older Adults: Pretest-Posttest Intervention Study Comparing Delivery Mode Preference and Attrition

**DOI:** 10.2196/13416

**Published:** 2019-08-28

**Authors:** Janet Maria Boekhout, Denise Astrid Peels, Brenda Angela Juliette Berendsen, Catherine Bolman, Lilian Lechner

**Affiliations:** 1 Department of Psychology and Educational Science Open University of the Netherlands Heerlen Netherlands

**Keywords:** older adults, physical activity, Web-based intervention, print-delivered intervention, attrition

## Abstract

**Background:**

Web-based interventions can play an important role in promoting physical activity (PA) behavior among older adults. Although the effectiveness of these interventions is promising, they are often characterized by low reach and high attrition, which considerably hampers their potential impact on public health.

**Objective:**

The aim of this study was to identify the participant characteristics associated with the preference for a Web-based or a printed delivery mode and to determine whether an association exists between delivery modes or participant characteristics and attrition in an intervention. This knowledge may enhance implementation, sustainability of participation, and effectiveness of future interventions for older adults.

**Methods:**

A real-life pretest-posttest intervention study was performed (N=409) among community-living single adults who were older than 65 years, with physical impairments caused by chronic diseases. Measurements were taken at baseline and 3 months after the start of the intervention. Hierarchical logistic regression was used to assess demographic and behavioral characteristics (age, gender, body mass index, educational attainment, degree of loneliness, and PA level), as well as psychosocial characteristics (social support for PA, modeling, self-efficacy, attitude, and intention) related to delivery mode preference at baseline and attrition after 3 months.

**Results:**

The printed delivery mode achieved higher participation (58.9%, 241/409) than the Web-based delivery mode (41.1%, 168/409). Participation in the Web-based delivery mode was associated with younger age (B=–0.10; SE 0.02; Exp (B)=0.91; *P*<.001) and higher levels of social support for PA (B=0.38; SE 0.14; Exp (B)=1.46; *P*=.01); attrition was associated with participation in the Web-based delivery mode (B=1.28; SE 0.28; Exp (B)=3.58; *P*<.001) and low educational attainment (B=–0.53; SE 0.28; Exp (B)=0.59; *P*=.049).

**Conclusions:**

A total of 41% of the participants chose the Web-based delivery mode, thus demonstrating a potential interest of single older adults with physical impairments in Web-based delivered interventions. However, attrition was demonstrated to be higher in the Web-based delivery mode, and lower educational attainment was found to be a predictor for attrition. Characteristics predicting a preference for the printed delivery mode included being older and receiving less social support. Although Web-based delivery modes are generally less expensive and easier to distribute, it may be advisable to offer a printed delivery mode alongside a Web-based delivery mode to prevent exclusion of a large part of the target population.

**Trial Registration:**

Netherlands Trial Register NTR2297; https://www.trialregister.nl/trial/2173

**International Registered Report Identifier (IRRID):**

RR2-DOI: 10.2196/resprot.8093

## Introduction

### Background

A majority of older adults in Western societies are not sufficiently physically active [1]. With societies ageing rapidly this poses a major public health concern, as insufficient physical activity (PA) is regarded as a major health risk. [2]. PA interventions that are cost effective in targeting this health risk are thus needed. Computer-tailored interventions designed to improve PA, delivered either via the Web or a printed delivery mode, have therefore become increasingly popular in the last decade, with substantial evidence supporting their effectiveness and cost-effectiveness [3-5]. However, computer-tailored interventions delivered on the Web may suffer from low reach (ie, the proportion of people participating in an intervention), low long-term adherence (ie, proportion of participants not following the intervention as intended), and high attrition (ie, the proportion of participants not completing the intervention) [6], thereby impeding the effectiveness of the interventions [7-9] and potentially decreasing their intended impact on public health. Web-based delivery modes can be especially challenging among older adults (older than 65 years). Current data on internet use among older adults show that despite the increasing popularity of Web-based interventions, a digital divide still exists [10], with only 50% of older adults regularly using the internet; a total of 34% of this age group express that they have little-to-no confidence to use the internet properly. Moreover, only 15% of adults older than 70 years use the internet for health applications [11,12]. Thus, alternative delivery modes should be provided for this population. Not only a higher age but also a lower educational attainment are characteristics of those on the disadvantaged side of the digital divide. Therefore, the individuals who are the most in need of health applications may not be reachable with interventions delivered via the Web [10]. Some physical limitations that older adults are confronted with, such as impaired eyesight, hearing, and dexterity, are inherent in this age group, and these may interfere with internet use in future generations of older adults [13]. Because offering interventions on the Web alone may exclude a vulnerable group, providing insight on how the (demographic and psychosocial) characteristics of older adults are associated with a preference for a printed or Web-based delivery mode and with attrition can contribute to stimulating reach and sustainability of such interventions. Moreover, it may help to explain the effectiveness of interventions and the impact on the public health of certain subpopulations [7,9,14].

Previously, the computer-tailored Active Plus65 intervention was developed with a primary aim to stimulate PA and a secondary aim to decrease loneliness [15]. The target population of Active Plus65 was single older adults with a physical impairment caused by a chronic disease, considering the high prevalence of insufficient PA and loneliness among this population [16-18]. Active Plus65 is available in 2 delivery modes, that is, Web-based (with both questionnaire and advice delivered through the internet) and printed (with a paper questionnaire and advice sent by paper post). Because interventions are often delivered in a single delivery mode, such as either on the Web or printed, it may be difficult to establish whether reach and attrition are related to the intervention itself or to its delivery mode. Apart from some technical applications, the content of the Web-based and printed versions of the Active Plus65 intervention was the same. This allowed a comparison of the Web-based and printed delivery mode of the computer-tailored Active Plus65 intervention.

### Objectives

There is a paucity of research determining the preferred and actually used delivery mode by really offering participants a choice in a real-life intervention setting. Previously, preferences have predominantly been researched by asking participants, hypothetically, what delivery mode they would prefer if they were to join an intervention [19-22]. As far as could be determined, there have been only 2 studies pertaining to delivery mode preference, where participants could choose between participating in a printed and Web-based delivery mode of an intervention [23,24]. In these studies, participants with lower education and older age chose the printed delivery mode. However, no straightforward comparisons can be made because the interventions in both studies were designed for different target groups compared with that of Active Plus65. However, the preference of a printed delivery mode in older and lower educated adults is also consistent with data on the present digital divide [25].

In 2012, the delivery mode preference and attrition of Active Plus50, a previous version of Active Plus65, were studied [26]. In Active Plus65, participants were free to choose between delivery modes, whereas participants in Active Plus50 were randomly assigned to either the printed or Web-based delivery mode. Active Plus50 participants in the Web-based delivery mode were younger, more often male, had a higher body mass index (BMI), and a lower intention to be physically active. Moreover, a low intention to be physically active was also found to be a predictor for attrition in both delivery modes. Because intention had a predictive value for delivery mode preference and attrition in Active Plus50, it may be interesting to repeat these analyses in Active Plus65: because participants could not freely choose their delivery mode in Active Plus50, the role of intention in Active Plus65, where participants can freely choose the delivery mode, may be different. Considering intention had a certain predictive value for delivery mode preference and attrition, other psychosocial variables, which according to several behavior change models are important determinants of intention [27-29], could also have a predictive value. Because low intention was encountered in the Web-based delivery mode, and it predicted attrition in both delivery modes, it can be argued that a negative attitude toward PA, low social support, modeling, and self-efficacy (psychosocial determinants associated with behavior change) may also be associated with a preference for the Web-based delivery mode and with attrition. After all, in behavior change theories, these are often the major determinants of behavioral intention that are strongly related to predicting behavior change. Such knowledge may be useful in the enrollment process of the intervention to ensure that participants join a delivery mode that is best suited for them; if, for example, our findings would establish a preference for a printed delivery mode in participants with low levels of social support, emphasizing on how easy it is to use the printed delivery mode or to point out that a helpdesk is available for Web-based participants at enrollment would be useful. Thus, participants with little social support who are hesitant about taking part in an intervention could be assessed with a short pre-enrollment questionnaire, thereby preventing them from not starting an intervention at all. Support for the incorporation of a broad range of (psychosocial) individual characteristics in the study can also be found in the Persuasion-Communication Matrix of McGuire [30]. This model describes several factors that predict whether an individual will use an intervention, among which the broad characteristics of the user are also included. For example, the approach required to stimulate a potential user with low self-efficacy and low social support to be physically active to join an intervention will vary from that for an individual with high self-efficacy and social support for health behavior. When trying to identify the characteristics that predict preference for a delivery mode, it may be interesting to focus on a broad range of determinants, including psychosocial determinants.

As Active Plus65 targets both PA and loneliness, it presents an opportunity to analyze whether the baseline level of PA and loneliness can predict delivery mode preference and attrition because this could link target subgroups to the most appropriate delivery mode. Only 2 studies have been found that considered PA to analyze delivery mode preference [20] or attrition [31]. In these studies, a higher level of PA was associated with a lower likelihood of preferring Web-based delivery modes and lower attrition. Comparisons may be difficult to make: either the Web-based delivery mode was not compared with a printed delivery mode but compared with face-to-face or group interventions instead [20] or only daily steps were measured [31] rather than a broad range of PA, which Active Plus65 does. Moreover, these studies were performed among a general population of adults. As far as could be determined, no previous research has considered loneliness when researching delivery mode and attrition of PA interventions for older adults; however, some assumptions may be made. In general, social isolation and loneliness are more prevalent among older adults [32,33]. Some studies have shown that social support is a prerequisite to adopt new technologies [34,35], which would suggest that participants who are lonelier will not choose a Web-based delivery mode. Considering the lack of studies for comparison, the analyses regarding PA and loneliness in our study will thus have a more exploratory character.

The aim of this study was to determine (1) which individual characteristics predict differences in delivery mode preference between the printed or Web-based delivery mode and (2) which user characteristics and delivery mode predict attrition. We hypothesized that a higher age, lower educational status, and lower presence of psychosocial determinants are predictors of a preference for a printed delivery mode and of attrition. Identifying the factors related to delivery mode preference and attrition could be of substantial use for researchers when optimizing the reach and sustainability of interventions because this could increase their impact on public health and prevent an important target population from being excluded when switching to only Web-based delivery prematurely.

## Methods

### Study Design

This study was part of a pretest-posttest trial, evaluating the Active Plus65 intervention [15,36,37]. The trial was executed in a real-life setting, without a control group. As the delivery mode preference and attrition of the participants in the printed and Web-based delivery mode of the intervention were compared, no control group was required for this study. This study was approved by the Research Ethics Committee of the Open University of the Netherlands (reference number U2016/02373/HVM). The original Active Plus50 studies were registered at the Netherlands Trial Register (NTR2297). All participants gave their informed consent before participation.

### Intervention

Active Plus65 is a computer-tailored intervention, with a primary aim to stimulate PA and secondary aim to decrease loneliness among single older adults with physical impairments caused by a chronic disease. Active Plus65 was systematically developed [15], and changes in PA [36] and loneliness [37] have been demonstrated.

**Figure 1:**
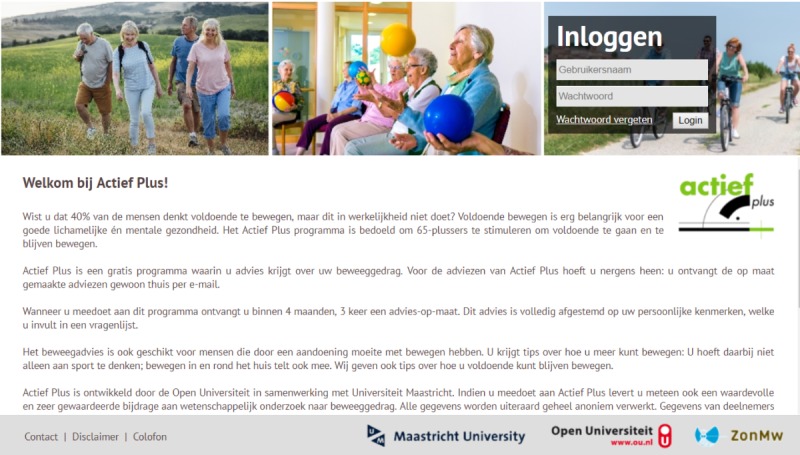
The intervention website.

The advice is generated by computer tailoring; in the Web-based delivery mode, participants fill the questionnaire themselves on the intervention website, which in the printed delivery mode is done by the intervention providers after receiving the questionnaire by mail from the participant. The method and degree of tailoring in both delivery modes is identical; therefore, the advice in both delivery modes has identical content, with only some practical differences. For example, in the printed version, modeling texts and pictures are used versus modeling videos in the Web-based version: the role models that are portrayed are the same persons delivering exactly the same message. Moreover, the design and format, such as images, typeface, and layout, of both questionnaires and advice are identical. A screenshot of the intervention website is provided in Figure 1.

**Figure 2:**
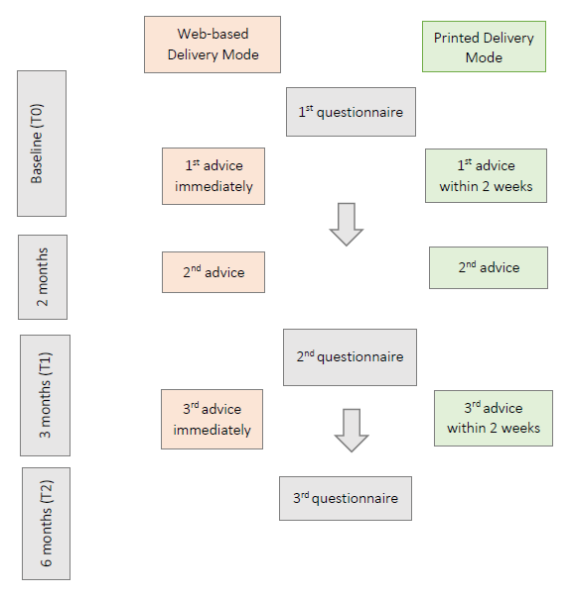
Intervention timelines per delivery mode.

Both delivery modes provide a tailored advice at 3 time points (at the start of the intervention, 2 months after the start, and 3 months after the start), on the basis of 2 questionnaires, the first one at the start (T0) of the intervention (on which advice 1 and 2 are based) and the second one after 3 months (T1; for advice 3). A third questionnaire, 6 months after the start of the intervention (T2), does not result in an advice, but it serves as a follow-up measurement. The time needed to fill out the questionnaire is identical for both delivery modes, that is, about 15 min for the first and second questionnaire and 5 min for the third questionnaire. Figure 2 provides a schematic view of the intervention timelines.

Each advice is tailored to the characteristics that are assessed in the questionnaire, including demographic and psychosocial determinants, the amount of PA, and the degree of loneliness. The first advice aims to increase insights into the present level of PA, which is achieved by targeting premotivational psychosocial determinants, such as knowledge and awareness. The second advice motivates participants to become more physically active and focuses on the benefits of PA, especially when done with others: in this advice, motivational psychosocial determinants, such as attitude, intention, and social influence, are targeted. Participants are also stimulated to prepare for difficult situations that might hinder them from becoming more active. The intervention also stimulates participants to transfer their motivation into sustainable behavior: depending on how active participants already are, this is done in the second or third advice by targeting postmotivational determinants, such as strategic planning and coping planning. Depending on the assessment, the advice comprises 7 to 12 pages (A4 format) of text, pictures, diagrams, etc, for (passive) reading (Multimedia Appendix 1) and elements that require participants to actively contribute, such as planning sheets that have to be filled, schemes for handling difficult situations and formulating implementation intentions, and basic PA exercises to be performed at home (Multimedia Appendix 2). The organization that implements the intervention (usually a local council) provides information on PA in social meeting opportunities that are available in the area where the participant lives. A more extensive description is provided elsewhere [15,36,37].

### Participants and Procedures

All citizens of a Dutch municipality, in the southern part of the Netherlands, who were single, older than 65 years, and living independently in the community (n=6751) were recruited by direct mailing. In this mail communication, it was explained that Active Plus65 is specifically suited for participants who have physical impairments caused by chronic diseases. For this study, only participants with a physical impairment were included. Invitations were sent by personalized letter and contained information about the intervention. Both log-in details for those wishing to participate via internet and a prepaid response card for requesting a paper questionnaire were included. It was mentioned that personal assistance with using internet when filling the questionnaire on the Web was available upon request. Only participants who completed the baseline questionnaire were enrolled. For the second assessment after 3 months, a printed invitation letter with the questionnaire and prepaid response envelope was sent to all participants who had completed the first questionnaire by the printed delivery mode. The participants in the Web-based delivery mode received an invitation by email for the follow-up questionnaires, with a direct link to the Web-based questionnaire. After 6 months, a third questionnaire was sent following the same procedure as for the second questionnaire.

### Measurements

Demographic characteristics, psychosocial determinants, the amount of PA, and the degree of loneliness were assessed at baseline. The second questionnaire assessed the same variables, except for the demographic characteristics considering their stable qualities. The third questionnaire assessed only the amount and type of PA and the degree of loneliness. Other variables were also assessed, but as they are outside the scope of this study, they are not discussed here.

#### Demographic Characteristics

The assessed demographic characteristics were age, gender, height, weight, educational attainment, and presence of physical impairments caused by chronic diseases. Height and weight were used to calculate the BMI by dividing weight in kilograms by height in meters squared. Educational attainment was categorized into *low* (lower vocational education, medium general secondary education, secondary vocational education, and higher general secondary education) and *high* (higher vocational education and university education). The presence of physical impairments was categorized into *yes* or *no*.

#### Psychosocial Determinants

The assessed psychosocial determinants were attitude, modeling, social support, self-efficacy, and intention to be sufficiently physically active. Attitude to be sufficiently physically active was measured by 17 items (eg, *PA gives me a satisfied feeling*) on a 5-point scale (1=totally disagree to 5=totally agree). Modeling was measured by asking *Are the following persons physically active for at least 30 min per day on at least 5 days per week?* in 2 items, 1 for family and 1 for friends, on a 5-point scale (1=never to 5=always). Social support for PA was also measured by 2 items (1 relating to family, 1 relating to friends) by asking *To what degree do you expect to get support to be sufficiently physically active?* —with answers on a 5-point scale (1=never to 5=always). Self-efficacy was measured by asking to what degree one would manage to be physically active for at least 30 minutes per day for different situations (eg, *when the weather is bad*), with 11 items on a 5-point scale (1=definitely not to 5=definitely sure). The intention for performing sufficient PA was measured by 3 items on a 10-point scale (eg, *How likely do you think it is that you will stay or become sufficiently physically active?*). The scales to assess the psychosocial variables were based on validated questionnaires [38-42], and their usability has been demonstrated by pilot tests among the target population [15].

#### Physical Activity

The amount of PA was assessed with the Short Questionnaire to Assess Health Enhancing Physical Activity (SQUASH) [43]. This questionnaire assesses the amount and intensity of different types of PA performed during (volunteering) work, commuting, household, and leisure time. It allows for calculating the total minutes per week of PA performed with moderate to vigorous intensity (MVPA) [2]. The psychometric properties of the SQUASH have been found to be acceptable [43-45].

#### Loneliness

Loneliness was assessed with the De Jong Gierveld 6-item Loneliness Scale, whose psychometric properties have been found to be acceptable [46]. This scale has 6 items (eg, *I often feel rejected*), on an originally 6-point scale, but it was adapted to a 10-point scale (1=*absolutely not* and 10=*absolutely sure*)*,* as this was deemed more suitable for older adults [47,48]. Items with answer ranges from 6 to 10 (indicating loneliness) are summed, resulting in a potential score of loneliness between 0 (not lonely) and 6 (extremely lonely).

### Statistical Analyses

#### Differences in Delivery Mode Preference

Statistical analyses were performed on those participants who completed the baseline questionnaire. Univariate one-way analyses of variance and chi-square tests were performed on age, gender, educational attainment, BMI, PA, loneliness, modeling, social support, attitude, self-efficacy, and intention to assess whether participants differed at baseline between the printed and Web-based delivery mode. Hierarchical logistic regression was performed on the T0 data to identify the user characteristics that predict differences in the preference for the printed or Web-based delivery mode. Outcome measure was the dichotomous variable of delivery mode. Step 1 of the analyses contained the demographic variables and the weekly minutes of MVPA and loneliness. In step 2, the psychosocial determinants were added. In step 3, interaction terms were added: as previous research provided no directions for formulating hypotheses on potential interaction effects, only interaction effects for the determinants that were significant in step 2 were included (eg, the interaction between age and social support).

#### Differences in Attrition

Differences in attrition between the printed and Web-based delivery mode were analyzed with a chi-square test. To identify potential factors related to attrition, hierarchical logistic regression analysis was performed on the T1 data, as this is the time when participants have to fill the second questionnaire, which will provide them with the third advice. Attrition occurs when participants do not fill this questionnaire. The outcome measure is the dichotomous variable of attrition. Demographic characteristics, weekly minutes of MVPA, and degree of loneliness were added in step 1; delivery mode in step 2; and psychosocial determinants in step 3 of the analysis. In step 4, interaction terms of user characteristics and delivery mode were added to assess whether dropout is associated with certain combinations of user characteristics and delivery mode: gender, age, and educational attainment were selected based on previous research [20,23,24].

All significance levels were set at *P*=.05, except for the final step of the regression analyses where significance was set at *P*=.10 because interaction terms are known to have less power [49]. SPSS version 24 (IBM Statistical Package for Social Sciences) was used to perform all analyses.

### Ethics Approval and Consent to Participate

The study was reviewed and approved by the Committee for Ethics and Consent in Research of the Open University (Commissie Ethische Toetsing Onderzoek, reference number: U2016/02373/HVM). Participants provided written informed consent to participate in the study.

## Results

### Delivery Mode Distribution

**Figure 3:**
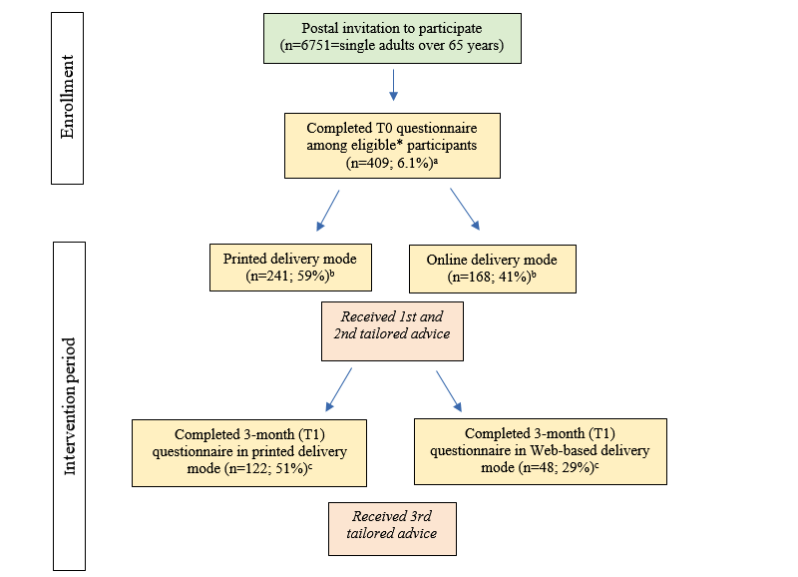
Flow chart of reach and attrition in printed and Web-based delivery mode. "a" reported as percentage of invited participants; "b" reported as percentage of all participants completing T0 questionnaire; "c" reported as percentage of all participants starting in either printed or Web-based delivery mode; asterisk indicates that eligible participants are those who meet all requirements of being single, over the age of 65, and chronically impaired in physical activity.

Of all eligible participants of Active Plus65, 241 (58.9%, 241/409) participants took part in the printed delivery mode, and 168 (41.1%, 168/409) took part in the Web-based delivery mode. In total, a response rate of 6% of invited participants was realized. Figure 3 provides an overview.

### Delivery Mode Preference

Several of the baseline characteristics of the printed and Web-based group differed significantly (Table 1).

Participants in the printed delivery mode were older (*P*<.001), had a lower educational attainment (*P*<.001), were more often female (*P*=.02), were less physically active (*P*<.001), had fewer family and friends who are sufficiently physically active as modeling roles (*P*<.001), received less social support for PA (*P*<.001), and had a lower intention to be sufficiently physically active (*P*=.01).

Baseline user characteristics related to delivery mode preference are presented in Table 2.

In step 1, age and degree of loneliness were significant predictors of delivery mode preference. Participants in the printed delivery mode were older (B=–0.10; SE 0.02; Exp (B)=0.91; *P*<.001) and lonelier (B=–0.14; SE 0.06; Exp (B)=0.87; *P*=.03) than the participants who chose the Web-based delivery mode. When entering the psychosocial variables to the analyses, age was still a significant predictor (B=–0.10; SE 0.02; Exp (B)=0.91; *P*<.001), but loneliness became nonsignificant (*P*=.16) and social support for PA then emerged as a significant predictor (B=0.38; SE 0.14; Exp (B)=1.46; *P*=.01) with participants in the Web-based delivery mode having higher levels of social support than those in the printed delivery mode. Explained variance (*R*^2^) in the steps ranged between 0.15 and 0.19. The interaction in step 3 between age and social support was not significant (*P*=.34), indicating that the effect of age on delivery mode preference did not differ depending on the degree of social support.

**Table 1:** Baseline characteristics of participants in printed and Web-based delivery mode.

Determinants	Printed (n=241)	Web-based (n=168)	*P* value
Age (years), mean (SD)	79.22 (7.59)	73.29 (6.61)	<.001
Body mass index (kg/m^2^), mean (SD)	27.01 (4.89)	27.82 (5.27)	.12
Educational attainment (low), n (%)	167 (70.8)	83 (51.2)	<.001
Gender (men), n (%)	74 (30.7)	71 (42.3)	.02
Moderate-to-vigorous physical activity, mean (SD)	387.72 (527.11)	606.51 (687.41)	<.001
Loneliness, mean (SD)	3.2 (1.98)	2.94 (2.06)	.21
Modeling, mean (SD)	2.55 (1.49)	3.07 (1.14)	<.001
Social support, mean (SD)	1.79 (1.27)	2.33 (1.07)	<.001
Attitude, mean (SD)	3.42 (0.57)	3.49 (0.59)	.28
Self-efficacy, mean (SD)	3.47 (0.98)	3.42 (0.96)	.58
Intention, mean (SD)	6.51 (1.61)	6.99 (1.73)	.01

**Table 2:** Hierarchical logistic regression to study whether user characteristics predict differences in delivery mode preference.

Determinants		Step 1 (*R*^2^=0.152)^a^				Step 2 (*R*^2^ =0.186)^a^			
		Exp (B)	B	SE	*P* value	Exp (B)	B	SE	*P* value
**First block^b^**									
	Age	0.91	–0.10	0.02	<.001	0.91	–0.10	0.02	<.001
	Gender^c^	0.79	–0.24	0.26	.36	0.71	–0.34	0.27	.21
	Education^d^	1.43	0.36	0.26	.17	1.32	0.28	0.27	.30
	Body mass index	1.01	0.01	0.02	.56	1.02	0.02	0.03	.48
	Moderate-to-vigorous physical activity	1.00	0.00	0.00	.40	1.00	0.00	0.00	.49
	Loneliness	0.87	–0.14	0.06	.03	0.91	–0.10	0.07	.16
**Second block^b^**									
	Modeling	—^e^	—	—	—	0.95	–0.05	0.13	.71
	Social support	—	—	—	—	1.46	0.38	0.14	.01
	Attitude	—	—	—	—	0.84	–0.17	0.28	.54
	Self-efficacy	—	—	—	—	0.77	–0.27	0.17	.11
	Intention	—	—	—	—	1.15	0.14	0.1	.19

^a^Explained variance (Nagelkerke *R*^2^).

^b^Printed coded 0, Web-based coded 1.

^c^Men coded 0, women coded 1.

^d^Low educational attainment coded 0, high educational attainment coded 1.

^e^Not applicable.

### Attrition

Attrition differed significantly between the delivery modes, that is, 50% in the printed delivery mode and 71% in the Web-based delivery mode (*P*<.001). Table 3 provides an overview of the predictors of attrition during the intervention.

The assessed demographic variables in step 1 were all nonsignificant. The delivery mode, added in step 2, was a significant predictor of attrition (B=1.34; SE 0.27; Exp (B)=3.81; *P*<.001), with attrition higher among participants in the Web-based delivery mode than in the printed delivery mode. By adding the psychosocial determinants to the analyses in step 3, delivery mode remained significant (B=1.28; SE 0.28; Exp (B)=3.58; *P*<.001) and educational attainment also became significant (B=–0.53; SE 0.28; Exp (B)=0.59; *P*=.049): attrition was higher among participants in the Web-based delivery mode and among participants with a low educational attainment than for those in the printed delivery mode and for participants with a high educational attainment. Explained variance (*R*^2^) in the steps ranged between 0.02 and 0.12. The interactions that were assessed in step 4 (ie, delivery mode x gender [*P*=.11], delivery mode x age [*P*=.22], and delivery mode x education [*P*=.26]) were all nonsignificant.

**Table 3:** Hierarchical logistic regression to study whether user characteristics, delivery mode, and interaction predict differences in attrition.

Determinants		Step 1 (*R*^2^=0.023)^a^				Step 2 (*R*^2^=0.101)^a^					Step 3 (*R*^2^=0.118)^a^				
		Exp (B)	B	SE	*P* value		Exp (B)	B	SE	*P* value		Exp (B)	B	SE	*P* value
**First block^b^**															
	Gender^c^	0.86	–0.16	0.25	.53		0.91	–0.09	0.26	.73		0.85	–0.16	0.27	.55
	Age	0.97	–0.03	0.02	.11		1.00	0.00	0.02	.92		1.00	0.00	0.02	.94
	Education^d^	0.70	–0.36	0.24	.14		0.61	–0.49	0.26	.06		0.59	–0.53	0.28	.049
	Body mass index	0.97	–0.04	0.02	.13		0.96	–0.04	0.02	.08		0.96	–0.04	0.03	.09
	Moderate-to-vigorous physical activity	1.00	0.00	0.00	.29		1.00	0.00	0.00	.18		1.00	0.00	0.00	.23
	Loneliness	0.94	–0.07	0.06	.26		0.97	–0.03	0.06	.64		0.98	–0.02	0.07	.73
**Second block^b^**															
	Delivery mode^e^	^___f^	—	—	—		3.81	1.34	0.27	<.001		3.58	1.28	0.28	<.001
**Third block^b^**															
	Self-efficacy	—	—	—	—		—	—	—	—		0.80	–0.22	0.16	.16
	Intention	—	—	—	—		—	—	—	—		0.99	0.00	0.10	.99
	Modeling	—	—	—	—		—	—	—	—		1.16	0.15	0.13	.23
	Social support	—	—	—	—		—	—	—	—		1.08	0.07	0.14	.60
	Attitude	—	—	—	—		—	—	—	—		1.11	0.11	0.27	.69

^a^Explained variance (Nagelkerke *R*^2^).

^b^Nonattrition coded 0, attrition coded 1.

^c^Men coded 0, women coded 1.

^d^Low coded 0, high coded 1.

^e^Printed coded 0, Web-based coded 1.

^f^Not applicable.

## Discussion

### Principal Findings

This study aimed to determine which user characteristics predict the preference for either a Web-based or printed delivery mode of a PA intervention for single older adults with physical impairments. In addition, this study examined which user characteristics and delivery mode predict attrition. This provides insights into which factors should be considered when designing PA interventions for this target population.

### Delivery Mode Preference

A total of 41% of the participants chose to start in the Web-based delivery mode. Although this demonstrated a potential interest of single older adults with physical impairments in Web-based delivered interventions, the majority still preferred a printed delivery mode. This is in agreement with data on the existing digital divide, showing that only 50% of older adults regularly use the internet, with only 15% using health applications [11-13]. These findings corroborate previous research and data suggesting that despite the increase in internet use among older adults over the last decade, it may still take many years for internet delivery mode to be the leading preference among all age groups [50-52]. Therefore, presently, intervention developers should not rule out printed delivery modes for this target population, as this could lead to the exclusion of a large segment of the target population.

Age was found to be a significant predictor of delivery mode preference, with older participants preferring the printed delivery mode more often. This finding is consistent with our hypothesis, as well as with previous research [23,24,26]. This finding is also corroborated by the Unified Theory of Acceptance and Use of Technology (UTAUT) [53]. In this model, performance expectancy, effort expectancy, and social influence determine usage intention, and through intention, they influence behavior. According to UTAUT, it may be that as older adults have less experience with the internet [54,55], they may expect the Web-based delivery mode of Active Plus65 to be more difficult and consequently choose the printed delivery mode; this preference may be enhanced by the social influence of peers who have the same expectations.

In contrast to our hypothesis, educational attainment was not found to be a significant predictor of delivery mode preference. It could be that the lower use of internet for health-enhancing interventions among people with a lower educational attainment [52,56-58] is outweighed by a general increase in availability and use of internet by older adults [54]. Another explanation is that assistance with internet offered when inviting participants for Active Plus65 could have given less educated participants enough confidence to participate in the Web-based delivery mode. In practice, only 10% of participants used this offer, but other assistance may have been received, such as that by the participants’ own social network. This explanation is supported by the review of Kampmeijer et al [59] who found support to be essential to give older adults the confidence to experiment with new technologies. Another explanation may lie in the overrepresentation of the female gender (65%) and low educational attainment (63%) at baseline. As educational opportunities have long been to the disadvantage of women, there’s a likelihood of educational attainment being less indicative of intelligence or digital literacy for older women; therefore, it may not have a predictive value for delivery mode.

In step 1 of the exploratory analyses into the predictive value of PA and loneliness on delivery mode preference, only loneliness was found to be a significant predictor: a higher degree of loneliness was found among participants in the printed delivery mode. In step 2 of the analyses, loneliness became nonsignificant, and social support for being physically active emerged as a significant predictor: participants with lower social support for being physically active preferred the printed delivery mode. Possibly, participants who receive less social support for being physically active also receive less social support for other aspects of life, such as for digitalization, thus making them less inclined to participate in a Web-based intervention. This is supported by previous studies that argue that those with close social support receive explanation and encouragement to use new technologies, such as internet, making it easier for them to adopt Web-based interventions [50,60,61]. Policy makers who strive to increase internet use within health care (eg, for budgetary reasons) should pay special attention to those who are older and have lower social support. Because these groups showed a preference for a printed delivery mode, steering them strictly toward internet delivery could risk losing them altogether for the intervention. For this subgroup, it may be essential to emphasize that the Web-based intervention is easy to use. In addition, future intermediaries of interventions could consider providing internet training opportunities to stimulate the use of internet-delivered interventions. A pre-enrollment questionnaire that assesses the level of internet literacy could be useful to determine the optimum format of the intervention.

### Attrition

Overall, attrition from the intervention was 58%. Although this is considerable, it is not uncommon: other studies on PA interventions for older adults have shown widely varying attrition rates, ranging from 22% to 76% [62-64]. However, several more recent studies have shown relatively low attrition rates (ranging from 0% to 51%, with a mean of 21%) [65,66], and these provide indications that an association between lower attrition and higher age may be present [26,66]. When considering these studies, the attrition from our intervention appears relatively high. Considering the relatively high attrition, for future research, a deeper analysis into the appreciation of the intervention would be useful.

Only delivery mode and educational attainment were found to be significant predictors of attrition: attrition was higher among participants in the Web-based delivery mode and among those with a lower educational attainment. The fact that only 2 determinants were found to predict attrition indicates that computer tailoring in Active Plus65 delivers advice that is equally valued in a broad range of participants. To provide corroboration for the finding that both the Web-based delivery mode and low educational attainment are predictors of attrition, 2 models can be outlined, that is, the Senior Technology Acceptance and Adoption Model (STAM) [67] and the Cycle of Technology Acquirement by Independent Living Seniors Model (C-TAILS) [68]. In STAM, the ease of learning is a crucial determinant for conversion to a new technology. It may be that the lower educated participants who took part in the Web-based delivery mode are unable to succeed in comfortably using the Web-based delivery mode and consequently stop using the intervention. In addition, C-TAILS stipulates that a new technology needs to be aligned with an individual’s needs: because Active Plus65 is not acquired on the participants’ initiative, it may be that their need for an intervention is lower and for the lower educated participants in particular, initial difficulties with using the Web-based intervention results in attrition. It may have some practical implications that attrition is higher in the Web-based delivery mode and among those with a lower educational attainment. For future intervention development, including targeted retention techniques specifically for Web-based delivery, such as email prompts, or delivering not all advice at once but in stages, could decrease attrition [14,69]. Several studies show that presentation strategies of interventions may need to be tailored considering those with low educational attainment to decrease attrition, for example, using more graphic materials instead of text and using entertaining or interactive elements [70,71].

It was also hypothesized that older age would be a predictor of higher attrition, but this was not established. An explanation may lie in the specific characteristics of our target population: participants who are older, have poor health status, and are unemployed will more often use an intervention as intended and will thus show lower attrition [72]. Those demographic determinants are comparable with the characteristics of the participants of Active Plus65 who are older, have physical impairments, and are mostly retired; although being retired may not be directly comparable with being unemployed, there are obvious similarities. Conversely, another characteristic specific to our target population, being single, may have had an opposite effect on attrition: it has been demonstrated that not having a life partner negatively influences internet use [50], which could contribute to a higher attrition rate.

Our assumption that a stronger presence of the psychosocial determinants associated with behavior change would be related to lower attrition was not confirmed. At 3 months in the intervention, participants already received advice 2 times. It could be that from this advice, participants obtain the anticipated aid they needed from the intervention and decide to discontinue use. That may even be more so in cases where higher levels of variables associated with behavior change are present: Active Plus65 focuses strongly on stimulating the motivation for PA, and there is a possibility that for participants who already have a higher commitment to behavior change, the additional value of Active Plus65 is less distinct. It has been suggested that especially in Web-based interventions, participants may stop using an intervention once they achieve outcomes they consider adequate [73]. Attrition from this point of view may not even be negative but rather be an affirmation of realizing what participants had expected to gain. This shows that a solid insight into the preintervention characteristics that are predictive of attrition may be useful before enrolling participants in the intervention. In line with that, more insight into the appreciation of the intervention could provide valuable information.

### Strengths and Limitations

As far as could be determined, this is the only study assessing the reach and attrition of the Web-based and printed delivery mode of an intervention with identical content among a population of single older adults with a physical impairment. As this population is growing fast, this study provides valuable insights. However, some limitations need to be acknowledged.

First, with 6%, the response rate appears to be quite low. Although a recent review showed that this is consistent with similar interventions [74], low response rates limit the public health impact of such interventions. The relatively low response rates and nonavailability of information on nonparticipants make it impossible to perform predictive analyses on who is interested in such interventions. We can only provide insight into the older adults who actually chose to participate. Second, only the baseline characteristics were included as potential predictors of attrition: other variables, such as digital literacy, engagement, or satisfaction with the intervention, may be related to attrition, but this could not be determined. Third, attrition from the intervention was relatively high, although this is not uncommon in eHealth interventions for older adults and in agreement with comparable studies [75]. Fourth, our study focuses on a specific subpopulation of older adults, that is, those who have a physical impairment and are single. As older age is generally accompanied by the onset of physical impairments, most older adults will meet this particular characteristic of our target population. However, this will not be applicable for the characteristic of being single, which may have implications for the generalizability of our findings. Considering the aim of our intervention, that is, stimulating PA preferably done with others, there’s a possibility that our intervention impacts singles and nonsingles differently. It may thus be advisable to repeat our studies in a population of mixed-marital status. Finally, the proportion of variance explained by our analyses appears relatively low (2%-19%), despite the inclusion of a broad range of potential demographic, health, and psychosocial determinants for delivery mode preference or for attrition. Nonetheless, these results are in line with comparable studies [23,26].

### Conclusions

The findings of our study outline which delivery modes are likely to be the most advisable for specific target populations, thus increasing the impact that interventions can potentially have on public health. Our results show that participants who are older and have lower levels of social support for PA are more attracted to the printed delivery mode of Active Plus65. Attrition was higher among those with a lower educational attainment, indicating that for these participants, print-delivered interventions would yield higher participation rates than Web-based delivered interventions. Although the Web-based delivery mode showed a higher attrition rate, printed delivery modes in general have the downside of being more expensive. It may therefore be advisable that printed delivery modes and Web-based delivery modes are offered alongside each other. Further research may also provide potential solutions to decrease attrition among those with lower education attainment. Considering the high speed at which changes in internet use occur, a continuous research into delivery mode preference and attrition is needed.

## Appendix

Multimedia Appendix 1 Advice that focuses on being active with a chronic disease.

Multimedia Appendix 2 Example of PA exercise done at home from emailed advice.

## Abbreviations

BMI: body mass index
C-TAILS: Cycle of Technology Acquirement by Independent Living Seniors Model
MVPA: moderate to vigorous physical activity
PA: physical activity
SQUASH: Short Questionnaire to Assess Health Enhancing Physical Activity
STAM: Senior Technology Acceptance and Adoption Model
UTAUT: Unified Theory of Acceptance and Use of Technology
