# Supplementary figures and images for: A Web-Based and Print-Delivered Computer-Tailored Physical Activity Intervention for Older Adults: Pretest-Posttest Intervention Study Comparing Delivery Mode Preference and Attrition
Source: J Med Internet Res. 2019 Aug 28;21(8):e13416. doi: 10.2196/13416 (PMC6737888; doi:10.2196/13416)

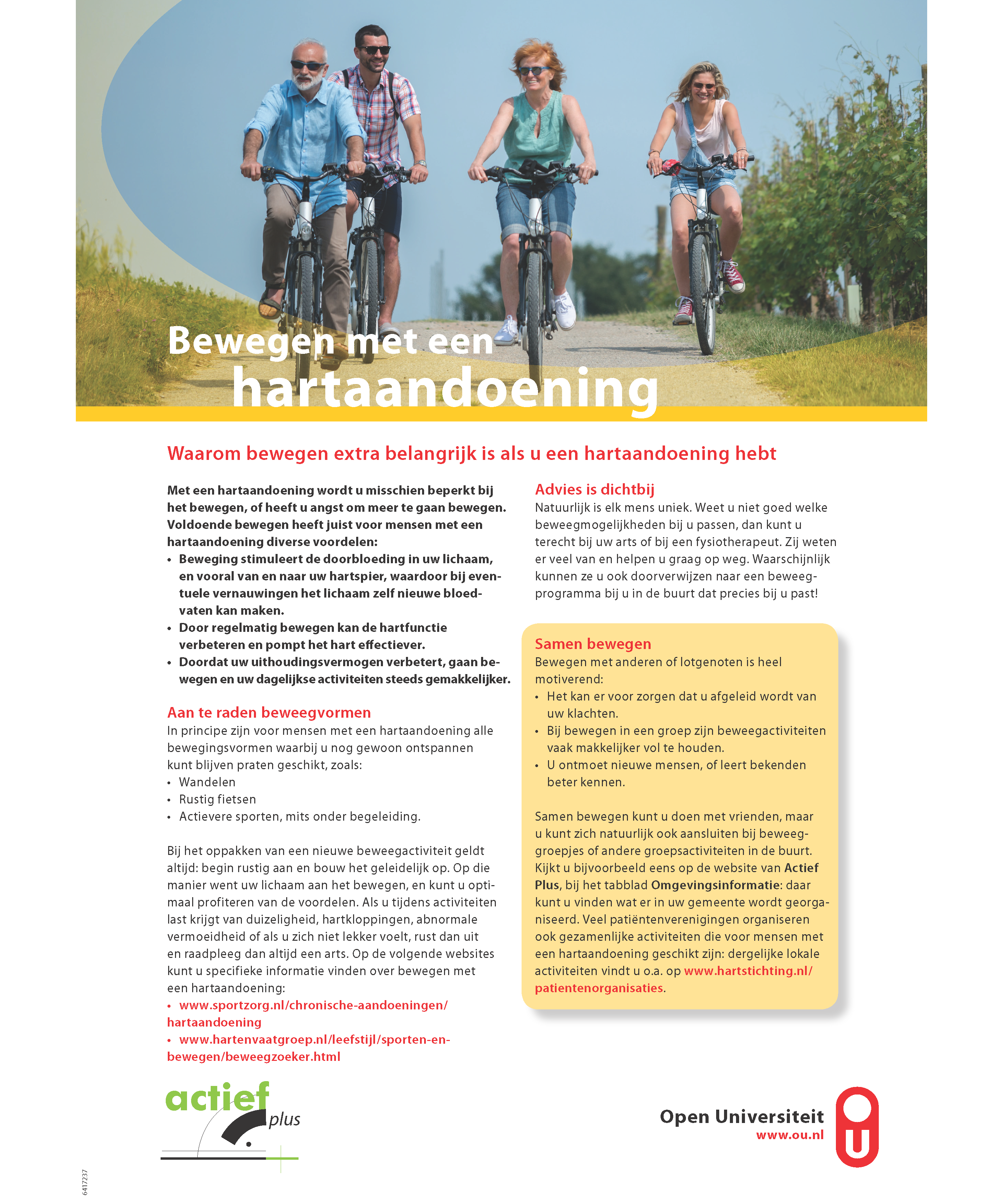

Supplement: Multimedia Appendix 1 [file jmir_v21i8e13416_app1.png]

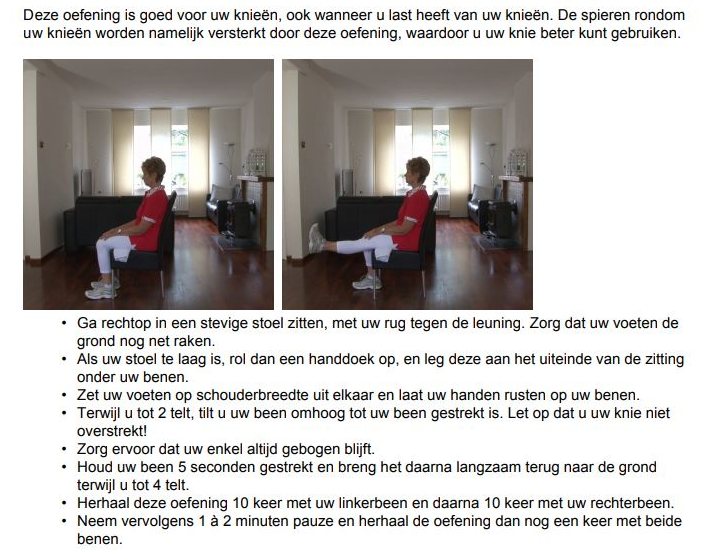

Supplement: Multimedia Appendix 2 [file jmir_v21i8e13416_app2.PNG]
